# Is It Time for Machine Learning Algorithms to Predict the Risk of Kidney Failure in Patients with Chronic Kidney Disease?

**DOI:** 10.3390/jcm10051121

**Published:** 2021-03-08

**Authors:** Charat Thongprayoon, Wisit Kaewput, Avishek Choudhury, Panupong Hansrivijit, Michael A. Mao, Wisit Cheungpasitporn

**Affiliations:** 1 Division of Nephrology and Hypertension, Department of Medicine, Mayo Clinic, Rochester, MN 55905, USA; charat.thongprayoon@gmail.com; 2 Department of Military and Community Medicine, Phramongkutklao College of Medicine, Bangkok 10400, Thailand; wisitnephro@gmail.com; 3 School of Systems and Enterprises, Stevens Institute of Technology, Hoboken, NJ 07030, USA; achoud02@syr.edu; 4 Department of Internal Medicine, University of Pittsburgh Medical Center Pinnacle, Harrisburg, PA 17105, USA; hansrivijitp@upmc.edu; 5 Division of Nephrology and Hypertension, Department of Medicine, Mayo Clinic, Jacksonville, FL 32224, USA; mao.michael@mayo.edu

Chronic kidney disease (CKD) is a common clinical problem affecting more than 800 million people with different kidney diseases [1]. CKD is associated with poor clinical outcomes including cardiovascular complications, a reduced quality of life, increased healthcare resource utilization and death [1,2]. In a few cases, CKD may progress to end-stage kidney disease (ESKD), leading to even higher morbidity and mortality [3]. The common, broadly used and readily relevant risk prediction tool with extensive validation is the kidney failure risk equation (KFRE) [3,4]. This equation includes age, sex, urine albumin-to-creatinine ratio (ACR) and estimated glomerular filtration rate (eGFR) to predict the need for kidney replacement therapy (KRT) including dialysis or kidney transplantation with excellent predictive performance in a Canadian population [4].

Subsequently, the KFRE equation has been externally validated in more than 30 countries [3,5,6,7,8,9,10]. Recently, Hallan et al. [10] studied the KFRE model in a Norwegian cohort of patients aged > 65 with an eGFR < 45 mL/min/1.73 m^2^, discovering it to be well-calibrated and maintaining a great discrimination (C-statistic of 0.93). This was comparable with the findings in a primary care setting in the United Kingdom [11]. Additional studies either validating the KFRE or implementing a similar approach to incorporate the KFRE have provided comparable findings including in a cohort in Korea (C-statistic of 0.86 in stage 3 CKD, 0.80 in stage 4 CKD and 0.84 in stage 5 CKD) [12], a cohort in Japan (C-statistic of 0.84 for a 3-variable model and 0.88 for an 8-variable model) [13] and a population in Singapore (0.93 for a 4-variable model) [14].

While the KFRE equation can provide excellent predictive performance, this predictive model does not take the effect of health-related behaviors on the risk of CKD progression into consideration [15]. In addition, the predicted risks for kidney failure by KFRE have been shown to be greater than the actual observed risks across the different etiologies of CKD with the exception of patients with polycystic kidney disease (PKD) [16]. Furthermore, each specific cause of CKD has additional important prognostic factors such as total kidney volume (TKV) in patients with PKD [17], immunosuppression in patients with glomerulonephritis [18] and the presence of rejection in kidney transplant recipients [19].

Recent progress in big data of electronic medical records (EMRs) has exponentially stimulated machine learning (ML) [1], which utilizes computer algorithms to identify patterns in big datasets with a large number of complex factors. ML can produce more precise prediction models by modeling linear and non-linear interactions among diverse and large numbers of variables, surpassing the capacity of traditional statistical approaches. Recent studies have demonstrated the potential application of ML approaches for CKD patients including the identification and monitoring of CKD [20]. With the advancement of natural language processing technology, clinical notes can also render an opening to discover previously unknown risk factors for CKD progression. While the data on the utilization of ML algorithms for CKD detection and monitoring are promising, data on prognostic algorithms to predict the risk of kidney failure in CKD patients are limited [20]. Recently, the Chronic Renal Insufficiency Cohort (CRIC) study investigators conducted a study utilizing an unsupervised ML algorithm with a consensus clustering on 72 baseline characteristics among 2696 CKD patients [21]. By utilizing a consensus clustering approach, the investigators successfully demonstrated distinct CKD subgroups that were associated with different risks of clinical outcomes [21]. This study’s findings support the promising direction towards the development of a ML risk prediction model for CKD progression. While the KFRE equation has already been validated and achieved an excellent predictive performance [3,5,6,7,8,9,10,11,13], ML approaches can further improve automatically through experience and the incorporation of more updated data in order to provide better healthcare and precision medicine [22]. In recent years, there have been significant advances in research on ML model interpretability and explainability; these have helped alleviate concerns of “black boxes” especially in neural network ML models [1].

In summary, this is a ripe time to investigate the use of ML approaches in nephrology. Its use in CKD patients would allow the development of ML models that better identify patients at risk of CKD progression and improve the management of CKD, especially in primary care settings.

## References

[1] Thongprayoon C., Kaewput W., Kovvuru K., Hansrivijit P., Kanduri S.R., Bathini T., Chewcharat A., Leeaphorn N., Gonzalez-Suarez M.L., Cheungpasitporn W. (2020). Promises of Big Data and Artificial Intelligence in Nephrology and Transplantation. J. Clin. Med..

[2] Vallabhajosyula S., Ya’Qoub L., Kumar V., Verghese D., Subramaniam A.V., Patlolla S.H., Desai V.K., Sundaragiri P.R., Cheungpasitporn W., Deshmukh A.J. (2020). Contemporary National Outcomes of Acute Myocardial Infarction-Cardiogenic Shock in Patients with Prior Chronic Kidney Disease and End-Stage Renal Disease. J. Clin. Med..

[3] Tangri N., Grams M.E., Levey A.S., Coresh J., Appel L.J., Astor B.C., Chodick G., Collins A.J., Djurdjev O., Elley C.R. (2016). Multinational Assessment of Accuracy of Equations for Predicting Risk of Kidney Failure: A Meta-analysis. JAMA.

[4] Tangri N., Stevens L.A., Griffith J., Tighiouart H., Djurdjev O., Naimark D., Levin A., Levey A.S. (2011). A predictive model for progression of chronic kidney disease to kidney failure. JAMA.

[5] Peeters M.J., van Zuilen A.D., van den Brand J.A., Bots M.L., Blankestijn P.J., Wetzels J.F. (2013). Validation of the kidney failure risk equation in European CKD patients. Nephrol. Dial. Transplant..

[6] Elley C.R., Robinson T., Moyes S.A., Kenealy T., Collins J., Robinson E., Orr-Walker B., Drury P.L. (2013). Derivation and validation of a renal risk score for people with type 2 diabetes. Diabetes Care.

[7] Marks A., Fluck N., Prescott G.J., Robertson L., Simpson W.G., Cairns Smith W., Black C. (2015). Looking to the future: Predicting renal replacement outcomes in a large community cohort with chronic kidney disease. Nephrol. Dial. Transplant..

[8] Lennartz C.S., Pickering J.W., Seiler-Mußler S., Bauer L., Untersteller K., Emrich I.E., Zawada A.M., Radermacher J., Tangri N., Fliser D. (2016). External Validation of the Kidney Failure Risk Equation and Re-Calibration with Addition of Ultrasound Parameters. Clin. J. Am. Soc. Nephrol..

[9] Whitlock R.H., Chartier M., Komenda P., Hingwala J., Rigatto C., Walld R., Dart A., Tangri N. (2017). Validation of the Kidney Failure Risk Equation in Manitoba. Can. J. Kidney Health Dis..

[10] Hallan S.I., Rifkin D.E., Potok O.A., Katz R., Langlo K.A., Bansal N., Ix J.H. (2019). Implementing the European Renal Best Practice Guidelines suggests that prediction equations work well to differentiate risk of end-stage renal disease vs. death in older patients with low estimated glomerular filtration rate. Kidney Int..

[11] Major R.W., Shepherd D., Medcalf J.F., Xu G., Gray L.J., Brunskill N.J. (2019). The Kidney Failure Risk Equation for prediction of end stage renal disease in UK primary care: An external validation and clinical impact projection cohort study. PLoS Med..

[12] Lee M.J., Park J.H., Moon Y.R., Jo S.Y., Yoon D., Park R.W., Jeong J.C., Park I., Shin G.T., Kim H. (2018). Can we predict when to start renal replacement therapy in patients with chronic kidney disease using 6 months of clinical data?. PLoS ONE.

[13] Hasegawa T., Sakamaki K., Koiwa F., Akizawa T., Hishida A. (2019). Clinical prediction models for progression of chronic kidney disease to end-stage kidney failure under pre-dialysis nephrology care: Results from the Chronic Kidney Disease Japan Cohort Study. Clin. Exp. Nephrol..

[14] Lim C.C., Chee M.L., Cheng C.Y., Kwek J.L., Foo M., Wong T.Y., Sabanayagam C. (2019). Simplified end stage renal failure risk prediction model for the low-risk general population with chronic kidney disease. PLoS ONE.

[15] Lee Y., Kwon S., Moon J.J., Han K., Paik N.J., Kim W.S. (2019). The Effect of Health-Related Behaviors on Disease Progression and Mortality in Early Stages of Chronic Kidney Disease: A Korean Nationwide Population-Based Study. J. Clin. Med..

[16] Hundemer G.L., Tangri N., Sood M.M., Ramsay T., Bugeja A., Brown P.A., Clark E.G., Biyani M., White C.A., Akbari A. (2020). Performance of the Kidney Failure Risk Equation by Disease Etiology in Advanced CKD. Clin. J. Am. Soc. Nephrol..

[17] Akbari A., Tangri N., Brown P.A., Biyani M., Rhodes E., Kumar T., Shabana W., Sood M.M. (2020). Prediction of Progression in Polycystic Kidney Disease Using the Kidney Failure Risk Equation and Ultrasound Parameters. Can. J. Kidney Health Dis..

[18] Suh J.S., Jang K.M., Hyun H., Cho M.H., Lee J.H., Park Y.S., Oh J.H., Kim J.H., Yoo K.H., Chung W.Y. (2020). Remission of Proteinuria May Protect against Progression to Chronic Kidney Disease in Pediatric-Onset IgA Nephropathy. J. Clin. Med..

[19] Chu C.D., Ku E., Fallahzadeh M.K., McCulloch C.E., Tuot D.S. (2020). The Kidney Failure Risk Equation for Prediction of Allograft Loss in Kidney Transplant Recipients. Kidney Med..

[20] Yang C., Kong G., Wang L., Zhang L., Zhao M.H. (2019). Big data in nephrology: Are we ready for the change?. Nephrology.

[21] Zheng Z., Waikar S.S., Schmidt I.M., Landis J.R., Hsu C.Y., Shafi T., Feldman H.I., Anderson A.H., Wilson F.P., Chen J. (2021). Subtyping CKD Patients by Consensus Clustering: The Chronic Renal Insufficiency Cohort (CRIC) Study. J. Am. Soc. Nephrol..

[22] Ahmed Z., Mohamed K., Zeeshan S., Dong X. (2020). Artificial intelligence with multi-functional machine learning platform development for better healthcare and precision medicine. Database.

